# Transition to Motherhood and Lived Experiences of Teenage Mothers Delivering in Kasese and Bundibugyo Districts, Western Uganda

**DOI:** 10.7759/cureus.63985

**Published:** 2024-07-06

**Authors:** Joseph Ngonzi, Brenda Ainomugisha, Onesmus Byamukama, Wilson Tumuhimbise, Josephine Asiimwe, Arnold Kamugisha, Moses Ntaro, Grace Nambozi, Lisa Bebell

**Affiliations:** 1 Obstetrics and Gynecology, Mbarara University of Science and Technology, Mbarara, UGA; 2 Information Technology, Mbarara University of Science and Technology, Mbarara, UGA; 3 Business Administration, Mbarara University of Science and Technology, Mbarara, UGA; 4 Community Health, Mbarara University of Science and Technology, Mbarara, UGA; 5 Nursing, Mbarara University of Science and Technology, Mbarara, UGA; 6 Medicine, Massachusetts General Hospital, Harvard Medical School, Boston, USA

**Keywords:** uganda, transition to motherhood, lived experiences, maternal child, teenage pregnancy

## Abstract

Introduction

A large number of teenagers in low-resource settings experience pregnancy, with a significant number of these cases happening in sub-Saharan Africa. Teenage pregnancy is associated with unique physical and psychological experiences.

Objective

To explore the lived experiences of teenage mothers delivering at a tertiary referral hospital in southwestern Uganda.

Methods

This qualitative study used focus group discussions (FGDs) with teenage mothers in Kasese and Bundibugyo districts in Uganda. We purposively sampled 32 teenage mothers attending a tertiary referral hospital who had been pregnant at least once and had given birth. Sociodemographic information was obtained, and FGDs were conducted to capture the teenagers’ experiences transitioning to motherhood. An inductive content analytic approach was used to analyze data.

Results

The mean age of the participants was 18.4 (standard deviation [SD], 1.2) years, with the majority (22, 68.8%) being rural dwellers, married (23, 71.9%), unemployed (21, 65.6%), and having attained primary education (23, 71.9%). Teenage mothers’ lived experiences were characterized by shattered dreams, concerns about changes in their body size and shape, abandonment and neglect by family members and spouses, considerations of terminating the pregnancy, forced early marriages, family stereotypes, and engaging in sex for survival. The process of transitioning to motherhood occurred along with resilience post-pregnancy and supportive environments from their loved ones, which enabled them to accept reality and care for their children.

Conclusions

The lived experiences of teenage mothers demonstrated social pressures, fear of abandonment, and poverty as major influences on their mindset and behavior. Further research to gain a comprehensive understanding of the challenges encountered by teenage mothers will aid the development of culturally appropriate strategies to reduce teenage pregnancy and improve perinatal outcomes.

## Introduction

Teenage pregnancy happens in those aged 10-19 years and is associated with many physical and psychological changes [1,2]. Approximately 21 million teenagers in low-resource settings worldwide become pregnant, and 50% of these pregnancies are unintended and result in an estimated 12 million births [3,4]. Sub-Saharan Africa accounts for half the global prevalence of teenage pregnancy [5,6].

Teenage pregnancy is a high-risk condition owing to maternal physiological and psychological immaturity coupled with insufficient sexual and reproductive knowledge [7,8]. Teenage pregnancy can cause major health and social challenges, resulting in negative consequences for the teenager and society [9-12]. Teenage pregnancy can lead to inability to engage in further education, early marriage, living in poverty, abandonment, and neglect [13-15]. However, teenage pregnancy may lead to an increased sense of responsibility and motivation to fulfill personal aspirations [16].

Researchers often categorize the multiple contributing factors for teenage pregnancy as sociodemographic, familial, cultural, and reproductive behaviors [17]. Factors associated with teenage pregnancy include living in rural areas, early marriage, low education level, lack of communication between parents and teenagers about sexual and reproductive health, and family history of teenage pregnancy [18-23].

Despite the findings in these prior studies, there is a paucity of knowledge on the lived experiences of teenage mothers as they transition to motherhood in resource-limited settings, such as Uganda, a country in sub-Saharan Africa with the highest global burden of teenage pregnancy. Research on issues affecting pregnant and parenting teenagers is still limited in scope and skewed geographically despite the large burden of adolescent childbearing in many African countries [24]. To fill the knowledge gap regarding teenage mothers’ lived experiences, we aim to establish and explore the lived experiences of teenagers giving birth in Uganda’s largely rural districts of Kasese and Bundibugyo as they transition to motherhood.

## Materials and methods

Study design and setting

In this narrative qualitative study, we employed focus group discussions (FGDs) of teenage mothers in Kasese and Bundibugyo districts in Uganda. The districts were chosen because the project looking at adolescent issues is anchored in Kasese and Bundibugyo, where adolescent pregnancy challenges are highest. Kasese is located along the equator in western Uganda, approximately 359 km west of Kampala, Uganda’s capital and largest city. Bundibugyo is also located in western Uganda, 380 km west of Kampala. The Democratic Republic of Congo borders both districts to the west.

In our study procedure, we considered teenage mothers as emancipated minors based on their pregnancy status. After obtaining their written informed consent, we obtained sociodemographic information. We then conducted FGDs to capture the lived experiences of the teenage mothers until we reached thematic saturation. We purposively recruited teenage mothers based on their willingness to provide relevant information about their lived experiences during their pregnancy and transition to motherhood. The recruitment was done by the adolescent liaison officers in the Kasese and Bundibugyo districts. We conducted the Bundibugyo district participant FGDs at the Bundibugyo District Hospital compound and the Kasese district participant FGDs at Bugoye Health Centre III. We chose the participants who met the inclusion criteria (had been pregnant before and given birth to either a still or live birth) from the network of teenagers that the community adolescent unit oversees in Bugoye Health Centre III and Bundibugyo District Hospital.

We recruited participants if they had been pregnant before and given birth to either a still or live birth. We conducted four FGDs, each composed of eight participants, which lasted between 60 and 90 minutes. A research team female nurse, a timekeeper who also operated a voice recorder, and another nurse who recorded notes moderated the discussions. The moderator was well-trained in conducting FGDs. FGDs focused on the lived experiences of the participants and their transition into motherhood. We conducted them in local languages: Rukonjo in Kasese and Lubwisi or Rukonjo in Bundibugyo. Trained research assistants digitally recorded and transcribed the FGDs. The same trained research assistants translated the FGDs into English. Following each FGD, JN, AK, GN, and WT reviewed the transcripts for quality and clarity.

Ethical considerations

We obtained ethical clearance from the Mbarara University of Science and Technology Institutional Review Board, with a reference MUST 09/05-17 and Uganda National Council of Science and Technology HS967ES. We also followed the ethical principles outlined in the Declaration of Helsinki for medical research involving human subjects.

Data management and analysis

For the FGDs, we used inductive content analysis to derive categories describing the lived experiences of teenage mothers during pregnancy. JN, AK, and WT reviewed and discussed all transcripts for content relevant to the lived experiences of the participants and how they transitioned into motherhood. Following an iterative process, WT assembled a codebook from the identified concepts. This involved the development and description of codes and the selection of corresponding illustrative quotes. JN and WT reviewed, discussed, and approved the final codebook. They used NVivo (version 11; QSR International) software for qualitative data management.

## Results

Participants’ demographic characteristics

The participants’ mean age was 18.4 years (SD = 1.2). The majority were rural dwellers (22, 68.8%), married (23, 71.9%), unemployed (21, 65.6%), and had attained primary education (23, 71.9%), as shown in Table 1.

**Table 1:** Demographic characteristics of the participants

Variables	n (%)
Age in years (mean ± SD)	18.4 (1.2)
Religion affiliation	
Catholic	19 (59.4%)
Protestant	11 (34.4%)
Pentecostal	1 (3.1%)
Muslim	1 (3.1%)
Residence type	
Rural	22 (68.8%)
Marital status	
Single	8 (25.0%)
Separated or divorced	1 (3.1%)
Married	23 (71.9%)
Persons live with	
Husband	23 (71.9%)
Parent	5 (15.6%)
Relative or friend	3 (9.4%)
Alone	1 (3.1%)
Employed	
No	21 (65.6%)
Level of education	
Primary education	23 (71.9%)
Currently attending school or in the last 6 months	
No	31 (96.9%)

Findings from the FGDs

Lived Experiences of Teenage Pregnant Mothers and Their Transition to Motherhood

Five themes emerged explaining adolescent mothers’ lived experiences: (i) emotional challenges and broken promises, (ii) perceptions about body changes, (iii) intimate partner relationship challenges, (iv) abortion considerations, and (v) financial pressure. The overarching theme of coping and support explained the transition to motherhood.

Emotional Challenges and Broken Promises

Three subthemes emerged under this major theme: broken promises, abandonment, and neglect. Participants reported that their partners neglected and abandoned them after becoming pregnant. They reported that their partners could not take care of their financial needs, and some spouses later abandoned the family home. The spouses did not keep the promises they had made to the teenage mothers. Others continued living in poor conditions, unable to afford basic needs such as rent, making it difficult for them to take care of their households or pay medical bills for their children. Some spouses chased the teenage mothers from their homes after getting pregnant, which left them with no financial support.

Perceptions About Body Changes

Participants reported that they were concerned about changing body sizes and shapes, especially as their pregnancy advanced. They reported increased body size and other changes, such as frequent urination, swollen feet, vomiting, and frequent fatigue.

Intimate Partner Relationship Challenges

Two subthemes emerged under marriage challenges: forced marriages and perceived difficulty in getting married. Some participants reported that family members pressured them to get married to the men who impregnated them. Some parents chased their teenage children from their homes after becoming pregnant, and the only option was to stay with the men who impregnated them, not necessarily out of love but out of necessity. For some teenagers, this experience later resulted in abandonment and neglect.

Abortion Considerations

Some participants considered terminating their pregnancies because of the responsibility of providing for their newborns and their experience that the men who impregnated them denied the pregnancies. Some reported receiving advice from their peers to terminate the pregnancy, an illegal procedure in Uganda (except on specific medical grounds).

Financial Pressure

Participants reported having early sexual encounters to get money to meet their physical needs or to get someone to take care of their necessities. They attributed this to their parents’ or guardians’ inability to provide essentials, such as soap, menstrual pads, money for upkeep at school, and school fees. When they received gifts or money from their boyfriends, the teenagers reported having sex with them in exchange for the gifts and eventually became pregnant.

Transitioning to Motherhood

One major theme and two subthemes emerged around the process of transitioning to motherhood. The major theme was coping and supportive mechanisms. Under this theme, the two subthemes were post-pregnancy resilience and loved ones’ supportive environment (family members and spouses), which enabled the teenagers to accept reality and care for their children.

Post-pregnancy Resilience

Participants reported moving on and accepting reality after learning that they were pregnant. They accepted they were going to become mothers and prepared for how they would care for their babies.

Supportive Environment

Some participants reported factors that helped them during their pregnancy, including psychosocial and moral support from family members and spouses, which encouraged them to look to the future and deliver their children. Some thought their family members would treat them badly upon learning that they were pregnant but were surprised at the type of support they received.

The participants’ significant statements, subthemes, and major themes are summarized in Table 2.

**Table 2:** Qualitative study findings

Participants’ significant statements	Subthemes	Major themes
My biggest problem was that the man could not even take care of me. When we met, he promised to give me everything, but when I got pregnant, he ran away. I was left with a lot of bitterness (mother, Bundibugyo, group 1).	Broken promises	Emotional challenges and broken promises
I was chased away from home, and there was no money for rent, enough food, and other needs. I feel abandoned (mother, Bundibugyo, group 2).	Abandonment	
Yes, I still face challenges because there has been no care since the man left me. The children normally fall sick, yet there is no money, and I am stressed (mother, Bundibugyo, group 1).	Neglect	
When I got pregnant, many changes began happening to my body, including increased weight, tiredness, and swelling of my face and feet. At five months, I began to feel funny movements in my tummy, and it was a scary feeling, but I later got used to it when I learned that it was the baby moving within the womb (mother, Kasese, group 1).	Physical body changes	Perceptions about body changes
As the pregnancy grew, I used to urinate more often than usual. I had to look for more clothes that I could fit into because some of my clothes could no longer fit. These changes were a whole new experience for me (mother, Bundibugyo, group 2).	Functional body changes	
My parents forcefully gave me to the man who impregnated me against my will. They reasoned that because I was pregnant, I was mature enough to get married to the man so that his family would give my parents some gifts and a dowry in exchange (mother, Bundibugyo, group 2). A stranger impregnated me, and my parents wanted me to get married to him, yet I did not know where he came from. So, my parents put pressure on me, and the neighbors started talking about me. I felt useless but had to bear the situation (mother, Kasese, group 1).	Forced marriages	Intimate partner relationship challenges
I have a child, but it’s hard to get a boyfriend because of the child, so getting married may be difficult for me because this is an added responsibility (mother, Kasese, group 2).	Perceived difficulty in getting married	
I wanted to abort because of the responsibilities around providing for the child. I also thought about and was fearful of the consequences and complications of abortion. I got the courage to carry the pregnancy to birth (mother, Bundibugyo, group 1).	Fear to abort	Abortion considerations
My close friends in the village advised me to go for an abortion. They told me that if I aborted the pregnancy early, people would not know that I was ever pregnant. I feared an abortion (mother, Kasese, group 2).	Peer pressure	
The reason why I had sex and ended up getting pregnant was a desire for material things. At times, I would want to buy something, yet I had no money. So I thought if I go in for boys and have sex with someone, I will get money and buy that thing (mother, Kasese, group 2).	Sex to meet physical needs	Financial pressure
My parents died, and I struggled to survive together with my siblings. I was introduced to prostitution to get money to take care of my siblings, my child, and me (mother, Bundibugyo, group 1).	Transactional sex	
Transition to motherhood		
I started planning how to be a young mother when I found out I was pregnant. I started thinking about how to be a good mother and how to care for the baby (mother, Bundibugyo, group 1).	Post-pregnancy resilience	Transitioning to motherhood
When I missed my periods, I did not worry because I thought it was normal. I went to school and started to be sickly, and later, they suspected that I was pregnant. They tested me, and it came back positive. The school authorities sent me home. When I told my parents, I was scared that they would harass me, but surprisingly they supported me through the pregnancy and even after delivery (mother, Kasese, group 2). After telling the man that I was pregnant, he started taking care of me. He even bought baby clothes at delivery. He is very supportive (mother, Kasese, group 2).	Supportive environment	

## Discussion

Five themes emerged explaining adolescent mothers’ lived experiences: (i) emotional challenges and broken promises, (ii) perceptions about body changes, (iii) intimate partner relationship challenges, (iv) abortion considerations, and (v) financial pressure. The overarching theme that explained the transition to motherhood was coping and support. Some participants reported psychosocial and moral support from family members and spouses.

In our study, we found that some parents or guardians, as well as the men responsible for the pregnancies, neglected or abandoned the teenage mothers before or during their pregnancy. Other researchers found neglect of teenage mothers by the persons closest to them, including their partners, resulting in emotional breakdown [14,15]. Broken promises from the persons responsible for the teenage mothers’ pregnancies compounded this abandonment. This may be because the partners, some of whom are also teenagers, do not understand that the transition to motherhood requires psychological, social, and physical adaptation [25], a responsibility the partners are not ready or able to assume. 

Some participants in our study reported that they dropped out of school after falling pregnant and did not return to school even after giving birth. Other studies reported similar findings among other teenage mothers who left school and did not return [15,26-28]. Pregnant teenagers often leave school once their pregnancy is visible [24]. On returning, they may face discrimination, ridicule, and isolation from peers and teachers [28]. Many are socially excluded, which can lead to low self-esteem, identity crisis, academic decline, substance use, truancy, and even fleeing home [29]. Despite a desire to return to school, many teenage mothers face hindrances, such as lack of childcare, financial burdens, lack of support, cultural barriers, and fear of community backlash [30,31].

Our findings also included statements from some teenage mothers about their forced early marriage, which may expose teenage mothers to unplanned pregnancies [31]. Other researchers have found an association between teenage pregnancy and early marriage [32-34], which may relate to parents’ belief that their teenager is now mature enough to be married. Furthermore, their family may benefit by receiving a dowry from the man’s family [34].

Some teenage mothers reported experiencing new physical and psychological changes regarding size, shape, and feeling. Physical changes, such as changing body sizes and shape, and psychological changes, such as emotional stress and low self-esteem, are commonly reported in studies of teenage pregnancy [1,2,13,35-37]. There can be a resultant psychosocial trauma and abortion considerations among these teenage mothers [38].

Some participants considered terminating their pregnancies because of the responsibility of providing for their newborn’s needs and their perception that the men who impregnated them abandoned them. Teenagers may consider abortion because they are afraid that parenting would limit their futures, or they believe they cannot provide for a child’s needs, or they anticipate being the target of societal stigma [39].

Finally, some teenage mothers reported coping and supportive mechanisms, including receiving psychosocial and moral support from family members and spouses during pregnancy and after delivery, which helped them cope with the pressure and challenges associated with pregnancy and motherhood. Other researchers have reported associations between positive motherhood experiences and good social support, which contributed to teenage mothers’ feelings of acceptance and optimism [40]. For some teenagers in other studies, pregnancy and early motherhood brought them closer to their families, particularly mothers and female siblings [40,41]. In another study of teenage mothers, researchers reported that although the mothers of pregnant teenagers were disappointed, they welcomed their daughters’ pregnancies, and their families embarked on flexible arrangements to care for and meet the mother and baby’s needs. Social support during pregnancy can alleviate emotional and physical pressures and help to improve the mother and newborn’s well-being [41,42].

Strengths of the study

In our study, we identified teenage mothers’ lived experiences and how they transitioned to motherhood in a largely rural, low-resource setting in sub-Saharan Africa, which provides an evidence base for establishing mechanisms to support teenagers as they make the transition to becoming mothers.

Recommendations

Healthcare providers and persons interacting with teenagers need to raise awareness about the importance of providing pregnant teenagers with necessary social support, including support from the baby’s father, the teenager’s parents, and the pregnant teenager’s friend networks. Healthcare providers should tailor social support interventions to meet individual needs because women’s needs may differ. Furthermore, there is an ongoing need to continue efforts to prevent teenage pregnancies by forming teenage peer support groups in Kasese and Bundibugyo districts. These support groups can be supervised by the adolescent units at the health facilities in the two districts.

Limitations of the study

Our study documents the lived experiences of teenage mothers in rural Uganda. We gleaned detailed information about their lived experiences, including the transition to motherhood, but we did not gather information on the perspective and experience of other key stakeholders, including the teenagers’ parents, teenage boys, and the men responsible for the pregnancies. Such information could help highlight the structural and behavioral factors influencing the concerns and decisions around pregnancy, attending school, marriage, and finances for pregnant teenagers.

Given the nature of our study’s design, which involved conducting it through FGDs, some of the teenagers may have been reluctant to share specific information in a group setting. Future researchers could address this limitation by also conducting in-depth interviews with the teenagers and other stakeholders. The results may not be generalizable to all settings because we conducted the study among teenagers in largely agrarian settings rather than urban areas.

## Conclusions

This study in Kasese and Bundibugyo districts revealed that teenage mothers experience emotional challenges and broken promises, perceptions about body changes, intimate partner relationship challenges, abortion considerations, and financial pressure. As they transition to motherhood, they report psychosocial and moral support from family members and spouses.
